# System Wide Analysis of the Evolution of Innate Immunity in the Nematode Model Species *Caenorhabditis elegans* and *Pristionchus pacificus*

**DOI:** 10.1371/journal.pone.0044255

**Published:** 2012-09-28

**Authors:** Amit Sinha, Robbie Rae, Igor Iatsenko, Ralf J. Sommer

**Affiliations:** Max Planck Institute for Developmental Biology, Department of Evolutionary Biology, Tübingen, Germany; The University of Chicago, United States of America

## Abstract

The evolution of genetic mechanisms used to combat bacterial infections is critical for the survival of animals and plants, yet how these genes evolved to produce a robust defense system is poorly understood. Studies of the nematode *Caenorhabditis elegans* have uncovered a plethora of genetic regulators and effectors responsible for surviving pathogens. However, comparative studies utilizing other free-living nematodes and therefore providing an insight into the evolution of innate immunity have been lacking. Here, we take a systems biology approach and use whole genome microarrays to profile the transcriptional response of *C. elegans* and the necromenic nematode *Pristionchus pacificus* after exposure to the four different pathogens *Serratia marcescens, Xenorhabdus nematophila, Staphylococcus aureus* and *Bacillus thuringiensis* DB27. *C. elegans* is susceptible to all four pathogens whilst *P. pacificus* is only susceptible to *S. marcescens* and *X. nematophila*. We show an unexpected level of specificity in host responses to distinct pathogens within and across species, revealing an enormous complexity of effectors of innate immunity. Functional domains enriched in the transcriptomes on different pathogens are similar within a nematode species but different across them, suggesting differences in pathogen sensing and response networks. We find translation inhibition to be a potentially conserved response to gram-negative pathogens in both the nematodes. Further computational analysis indicates that both nematodes when fed on pathogens up-regulate genes known to be involved in other stress responses like heat shock, oxidative and osmotic stress, and genes regulated by DAF-16/FOXO and TGF-beta pathways. This study presents a platform for comparative systems analysis of two nematode model species, and a catalog of genes involved in the evolution of nematode immunity and identifies both pathogen specific and pan-pathogen responses. We discuss the potential effects of ecology on evolution of downstream effectors and upstream regulators on evolution of nematode innate immunity.

## Introduction

The struggle against infectious diseases caused by bacteria, viruses, fungi, protozoa and metazoan parasites is an important evolutionary agent [1] leading to rapid evolutionary changes responsible for much of the complexity found in the immune system of animals [2]–[4]. However, the molecular basis for the evolution of such host-pathogen interactions is only poorly understood. Over the past ten years studies of the nematode *Caenorhabditis elegans* have given insight into genes essential for host immunity [5], [6] as well as identifying bacterial virulence mechanisms used by opportunistic mammalian pathogens [7], [8]. These studies (and many others) have identified various signaling pathways critical for *C. elegans* survival when fed an array of bacterial and fungal pathogens e.g. ERK MAP kinase, p38 MAP kinase, TGF β, programmed cell death, DAF–2/DAF–16 insulin-like receptor signaling and JNK-like MAP kinase [6], [9]–[13], as well as components such as the G-protein coupled receptor FSHR-1, bZIP transcription factor *zip−2* and beta-Catenin/*bar−1* which are required for an inducible pathogen response [14]–[16]. It remains to be discovered however, how important these pathways are in other nematode species and how these pathways contribute to the evolutionary trajectories of bacterial pathogenicity. A comparative approach with another nematode species would provide a first entry point to enhance our understanding of the evolutionary diversity of host (nematode) response to pathogens.

One nematode that has been used extensively for comparative studies with *C. elegans* is the diplogastrid species *Pristionchus pacificus* (see [17]). In addition to having a well characterized proteome and a fully sequenced genome [18], [19], forward and reverse genetics [20], and transgenic techniques [21], full genome microarray technology has also been developed [22] allowing genomic analysis of many different traits. *P. pacificus* diverged from *C. elegans* 250–400 million years ago [18] and during this time there have been remarkable changes in vulva development [23], [24], gonad morphogenesis [25], sex determination [26] and chemosensory behaviour [27] allowing for evolutionary and developmental comparisons with *C. elegans*. These two nematodes also differ in their ecological niches. *C. elegans* can be isolated from compost heaps, snails and rotten fruits [28], whereas *P. pacificus* is usually isolated from a range of scarab beetles [29]–[32]. *P. pacificus*, as well as other *Pristionchus* species live in a necromenic lifestyle, that is feeding on microorganisms growing on the carcass of beetles once they die [29].

*C. elegans* and *P. pacificus* not only live in different ecological niches, but also differ in their susceptibility to bacterial pathogens. For example, *C. elegans* dies when fed the human opportunistic bacteria *Pseudomonas aeruginosa*, *Staphylococcus aureus* and insecticidal *Bacillus thuringiensis* Cry 5B toxin whereas *P. pacificus* is resistant [33], [34]. More recently, a screen of about 1,400 naturally strains of *Bacillus* yielded three strains of *Bacillus thuringiensis* DB27 that are extremely toxic to *C. elegans* but *P. pacificus* remain resistant [35]. Anatomically, *C. elegans* and *P. pacificus* differ in that *C. elegans* contains a grinder in the posterior bulb of the pharynx that is involved in the physical lysis of bacterial food [36]. While the grinder is a typical structure of nematodes of the Rhabditidae family, no grinder exists in nematodes of the Diplogastridae family, to which *P. pacificus* belongs [35]–[37]. Given these strong differences in the ecology and anatomy of *C. elegans* and *P. pacificus*, these two species represent ideal candidates for studying the evolution of the genetic mechanisms of pathogen response in nematodes.

Here, we used a systems level approach by testing in parallel four different bacterial pathogens that cause distinct effects on the two nematodes. We analyzed whole genome gene expression of *C. elegans* and *P. pacificus* when fed four different pathogens (*Serratia marcescens*, *Xenorhabdus nematophila*, *B. thuringiensis* DB27 and *S. aureus*) and compared each nematode pathogen response to those fed on the control bacterium (the standard nematode lab food *Escherichia coli* OP50). *S. marcescens* is a broad host pathogen present in soil and insects that kills *C. elegans* [8], [38] and *P. pacificus*. *X. nematophila* is a symbiotic bacteria of the entomopathogenic nematode *Steinernema carpocapsae*, which utilizes the bacteria to kill insects and feed on the resulting mass of proliferating bacteria [39], and also kills both the nematodes [34]. Our *B. thuringiensis* DB27 strain was isolated from a dung beetle (*Geotrupes* spp.) and seems to be one of the most pathogenic bacteria of *C. elegans* reported in the literature so far, which kills *C. elegans* in less than sixteen hours while *P. pacificus* is resistant [35]. We show an unexpected level of specificity in host responses to distinct pathogens within and across species, revealing an enormous complexity of effectors of innate immunity. This study presents (i) a platform for comparative systems biology of two nematode models, (ii) a catalog of genes involved in the evolution of nematode immunity and (iii) pathogen specific and pan-pathogen responses from both *C. elegans* and *P. pacificus*.

## Results and Discussion

### Survival of *C. elegans* and *P. pacificus* differs when fed gram-positive pathogens

**Figure 1:**
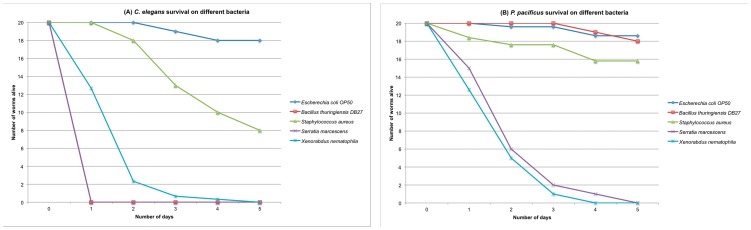
Differences in survival of *C. elegans* and *P. pacificus* upon exposure to different pathogenic bacteria. The survival of *C. elegans* and *P. pacificus* is different upon exposure to different bacteria. The survival curves for (A) *C. elegans* and (B) *P. pacificus* were obtained after exposure to the gram-positive bacteria *Bacillus thuringiensis* DB27 and *Staphylococcus aureus*, and the gram-negative bacteria *Serratia marcescens* and *Xenorhabdus nematophila*. Standard lab food *Escherichia coli* OP50 was used as a control for both nematodes. Both nematodes show reduced survival on *S. marcescens* and *X. nematophila*. *C. elegans* is also susceptible to *B. thuringiensis* DB27 and *S. aureus*, while *P. pacificus* shows higher resistance to these two bacteria.

To study the evolution of the genetic mechanisms involved in nematode resistance against bacteria, we fed the four bacterial pathogens *S. aureus*, *B. thuringiensis* DB27, *S. marcescens* and *X. nematophila* to the two nematode model species *C. elegans* and *P. pacificus* and assessed their effect on survival. When *C. elegans* is fed monoxenic lawns of each of the four pathogens, it dies within 2–5 days (Median survival time <5 days, Figure 1A). This is in stark contrast to *P. pacificus*, which is more resistant to the gram-positive pathogens *B. thuringiensis* DB27 and *S. aureus* and can survive for more than 7 days (Figure 1B and Figure S1, Median survival times ∼ 8 days). However, *P. pacificus*, like *C. elegans*, is susceptible to both *X. nematophila* and *S. marcescens* and 50% of the population dies within 2–3 days exposure (Figure 1B). We would like to note here that the difference in susceptibility of *P. pacificus* to gram-positive bacteria tested in this study is not simply an artifact of a longer life-span, as its wild-type life-span and developmental rate is comparable to that of *C. elegans* ([17], [40] and our unpublished observations). Also, *P. pacificus* is highly susceptible (median survival ∼ 3.5 days) to a gram-positive *Bacillus* strain DB35 isolated from *Geotrupes sp.* beetles [35], indicating that it is not more resistant to gram-positive bacteria in general. We also note that *P. pacificus* is able to survive and reproduce on both *B. thuringiensis* and *S. aureus* indicating that its reduced susceptibility should not be due to reduced bacterial intake.

### Significant transcriptional changes after exposure to different bacteria

To investigate the transcriptional response of the two nematodes upon exposure to these different bacterial pathogens, we identified differentially expressed genes using whole genome microarrays containing ∼43,000 probes for 20,149 *C. elegans* genes and ∼90,000 probes for 20,987 genes in *P. pacificus* respectively [22]. For each condition, total RNA was collected from four separate biological replicates of about 200 synchronized young adult worms exposed either to the pathogen or to the control *E. coli* (OP50) for 4 hours. The labeled cRNA produced from this total RNA was hybridized to species-specific microarrays according to manufacturer's protocols, and the raw data from scanned images was analyzed using the “limma” package in Bioconductor (see Methods for details). We observed that the exposure to pathogen resulted in a decrease of amount of total RNA produced per worm when compared to the relatively non-pathogenic *E. coli* strain (Figure S2). This global decrease in transcription is most likely a common feature of a core stress response, as it is also seen in case of dauer formation in both the species [22], [41] or might be an effect of the various bacteria on efficieny of RNA extraction. Nonetheless, such global transcriptional changes call for optimization of parameters used in normalization of microarray data, without which the calculated fold-changes can be erroneous [22], [42]. Our fold-change calculations take these factors into account (see Methods for details).

Although previous studies of pathogen response in *C. elegans* [9], [43]–[47] have looked at the transcriptome at different time-points such as 4-hours, 8-hours or 24-hours after exposure, we chose to investigate one of the earliest time-point of 4 hours because we were interested in earliest transcriptional responses manifested in response to each of these pathogens. Pathogenesis related necrosis of host-tissue at later time-points is a common effect of many pathogens and such organism-wide necrosis might dominate the expression profile at later time points [46], masking the pathogen specific signatures. Also, pathogens like *Bacillus thuringiensis* DB27 kill *C. elegans* in less than 24 hours, making analysis of late time-points unfeasible [35].

Widely different numbers of genes are found to be up- or down-regulated in *C. elegans* or *P. pacificus* upon exposure to different pathogens (Table 1A and 1B), indicating both a pathogen-specific and a nematode-specific component to these responses. In this context it is interesting to note that just a 4-hour exposure to pathogen is sufficient to cause large transcriptional changes in both the species, suggesting rapid activation of innate immune response.

**Table 1:** Widely different numbers of genes are differentially expressed in (A) *C. elegans* and (B) *P. pacificus* in response to the four bacteria.

(A) C. elegans	Up	Down	TOTAL	%Up	%Down
*B. thuringiensis*	5532	156	5688	97%	3%
*S. aureus*	181	68	249	73%	27%
*S. marcescens*	1465	4931	6396	23%	77%
*X. nematophila*	732	7884	8616	15%	85%

(B) P. pacificus	Up	Down	TOTAL	%Up	%Down
*B. thuringiensis*	156	61	217	72%	28%
*S. aureus*	178	140	318	56%	44%
*S. marcescens*	192	1007	1199	16%	84%
*X. nematophila*	848	4293	5141	16%	84%

The genes were called differentially expressed on microarrays if the FDR corrected p-value was less than 0.05 and the absolute value of fold changes was greater than 1.42 (corresponding to log2 fold change of 0.5 where log2(1.42) = 0.5).

### Changes in nematode gene expression depend on lethality and rate of killing

Based on the absolute number of differentially expressed genes under different conditions of survival, lethality and slower or faster killing rates of pathogens, some patterns can be discerned in our microarray data. First, the number of differentially expressed genes seems to be inversely correlated with the survival characteristics of the nematodes. For example, upon exposure to *B. thuringiensis* DB27, the pathogen most lethal to *C. elegans*, a remarkably large number of genes are affected in *C. elegans* (n = 5868, Table 1A), whereas much fewer genes (n = 217, Table 1B) are induced in *P. pacificus,* which is resistant to this pathogen. This can be attributed to the fact that while *P. pacificus* can use *B. thuringiensis* DB27 for food, *C. elegans* has to mount a robust response against a lethal pathogen.

For *C. elegans*, the number of differentially expressed genes is greater when exposed to faster-killing pathogens *B. thuringiensis* DB27, *X. nematophila* and *S. marcescens* as compared to that on *S. aureus*, where worms survive longer (Table 1A). Similarly, in *P. pacificus*, greater number of genes is differentially expressed on more lethal pathogens *X. nematophila* and *S. marcescens* (Table 1B) as compared to that on *B. thuringiensis* DB27 and *S. aureus* (Table 1B), to which *P. pacificus* is more resistant (Figure 1B).

Further, in *P. pacificus*, which is either more resistant to pathogens or shows slower mortality kinetics as compared to *C. elegans*, the expression profiles are observed to be usually smaller or just as large as that in *C. elegans*. We can rule out that these differences in profile size are due to potential differences in sensitivity of the two microarray platforms used, because we know from previous studies that our *P. pacificus* microarrays could detect differential expression of larger number of genes under different conditions such as dauer formation [22]. We also checked if changing the p-value cutoffs on microarray data abolishes the difference in profile sizes, but we find that the trend still holds (data not shown). Also, in *C. elegans* we have observed that when it is exposed to a non-pathogenic *Bacillus subtilis* strain for 4 hours, the number of genes differentially expressed is relatively low (∼510 genes) [II, AS and RJS, unpublished observations]. Hence, the differences in profile size between *C. elegans* and *P. pacificus* are most likely biologically relevant and not just a technical artifact.

### Gram-positive bacteria predominantly induce over-expression of genes while gram-negative bacteria cause transcriptional suppression

**Figure 2:**
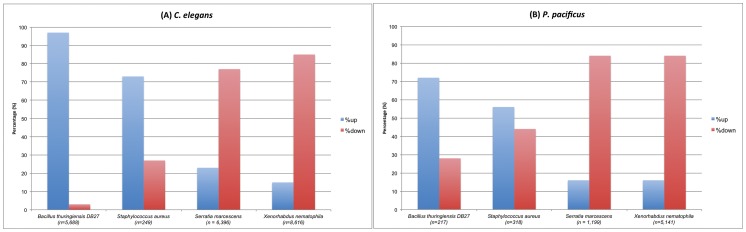
Gram-positive and gram-negative bacteria induce different proportions of up- versus down-regulated genes in *C. elegans* and *P. pacificus*. Despite the differences in number of genes differentially expressed on each of the pathogens in the two nematodes, the relative proportion of up-regulated genes is higher than that of down-regulated genes on gram-positive bacteria *B. thuringiensis* and *S. aureus* in both (A) *C. elegans* and (B) *P. pacificus*. On the other hand, exposure to the gram-negative bacteria *S. marcescens* and *X. nematophila* results in down-regulation of a greater fraction of genes as compared to the up-regulated genes in both (A) *C. elegans* and (B) *P. pacificus*.

For both nematode species, the gram-positive bacteria tested induce up-regulation of relatively more genes as compared to down-regulation, while the reverse seems to be true for the gram-negative bacteria (Table 1). For example, in both the nematodes, exposure to the gram-positive pathogens *B. thuringiensis* DB27 and *S. aureus* causes induction of relatively more genes as compared to suppression (Figure 2, Table 1). On the other hand, exposure to the gram-negative pathogens *S. marcescens* and *X. nematophila* predominantly causes down-regulation of comparatively more genes than up-regulation (Figure 2, Table 1). Thus, the relative proportion of up-regulated versus down-regulated genes appears to depend upon some common factor(s) shared by either the gram-negative or the gram-positive bacteria, although more bacteria from both groups need to be tested to confirm this trend.

### A bacterium-specific transcriptional response is mounted by both *C. elegans* and *P. pacificus*

**Figure 3:**
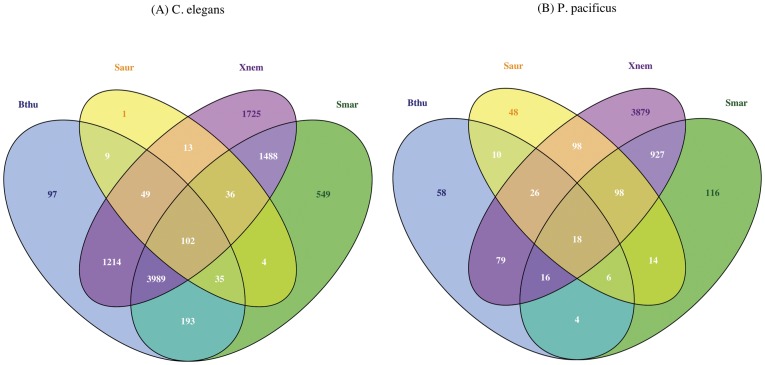
A pathogen-specific transcriptional response is mounted by both *C. elegans* and *P. pacificus* upon exposure to different bacteria. Overlap between the different genes differentially regulated in response to the four pathogens tested is represented as a Venn diagram for *C. elegans* and *P. pacificus*. Only 102 genes are found to be common between the expression profiles on all four pathogens in (A) *C. elegans*, while only 18 genes are common between the expression profiles corresponding to the four pathogens in (B) *P. pacificus*. This minimal overlap indicates the existence of a highly pathogen-specific immune response in both the nematodes. The abbreviations Bthu, Saur, Smar and Xnem refer to the bacteria *Bacillus thuringiensis*, *Staphylococcus aureus*, *Serratia marcescens* and *Xenorhabdus nematophila* respectively.

Next, we investigated the intra-specific response of the two nematodes after exposure to different pathogens. Although *C. elegans* is unable to survive on any of the four pathogens, we find that the expression profiles on each of the pathogens are qualitatively quite different from each other and only a small fraction of genes are common between expression profiles obtained on different pathogens (Figure 3A). Specifically, only 102 genes change their expression upon exposure to each of the four pathogens (Figure 3A, genes with nCommon  = 4 in Table S1) but the number slightly increases to about 687 genes when the criterion is relaxed to significant differential expression in more than one expression profile (genes with nCommon >1 in Table S1). A small number of overlap between multiple pathogen response profiles is a signature for highly specific pathogen response, and has also been observed before e.g. only 22 genes were reported to be common between profiles after 24 hour exposure to *Erwinia carotovara*, *Enterococcus faecalis*, and *Photorhabdus luminescens* [46]. Interestingly, the genes induced in *C. elegans* across all four pathogens include the transcription factors *pqm−1* and *zip−2* (Table S1). The stress responsive transcription factor *pqm−1* is also induced and required for defense in response to *P. aeruginosa* infection [43]. The bZIP transcription factor *zip−2* is a known to regulate a subset of *pmk-1* independent pathogen response genes on *P. aeruginosa* [15]. Similarly, the *P. pacificus* expression profiles also show a bacterium-specific signature, with only 18 genes being common across all the four profiles (n = 18, Figure 3B, genes with nCommon  = 4 in Table S2) while 206 genes are common between the expression profiles on more than one pathogen (genes with nCommon >1 in Table S2).

This pathogen specific nature of expression profiles within a nematode species is further highlighted in an expression cluster analysis (see Methods), where we compare our data-sets with various published microarray studies of pathogen-response [12], [43]–[50]. Based on the significance of overlaps between different microarray data sets (Table S3A and Table S4A for *C. elegans* and *P. pacificus* respectively), it is evident that only a small proportion of genes in each expression profile show an overlap with expression profiles on other pathogens.

**Figure 4:**
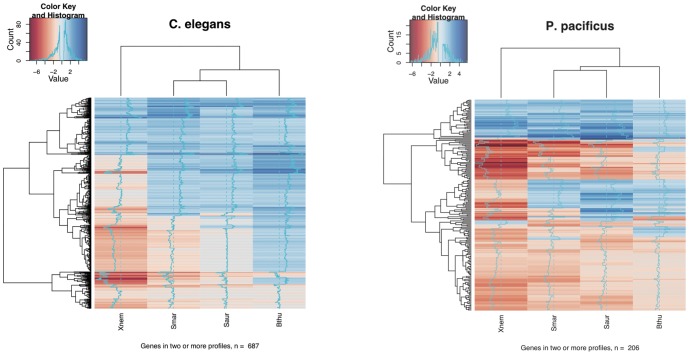
Hierarchical clustering of genes differentially expressed on more than one pathogen identifies clusters of co-regulated genes. The pathogen-response expression profiles for each nematode were clustered based on log2 of fold-changes for genes that were differentially expressed on at least two pathogens. In both (A) *C. elegans* and (B) *P. pacificus*, the expression profile in response to *X. nematophila* clusters separately from that in response to other pathogens, suggesting a difference in its mode of virulence from other pathogens.

We further evaluated the extent of similarities between different intra-specific expression profiles, by carrying out a two-dimensional hierarchical clustering [51] on log-fold change values of genes that were significantly differentially expressed on at least two bacterium (n = 687 genes in *C. elegans*, and n = 206 genes in *P. pacificus*, number of common 1∶1 orthologs  = 15, Table S1 and Table S2). Interestingly, in both *C. elegans* and *P. pacificus* heat-maps (Figure 4), the expression profile of *X. nematophila* response clusters separately from the profiles obtained in response to the other three bacteria, suggesting some differences in its pathogenicity mechanism(s) compared to other three bacteria. In summary, both *C. elegans* and *P. pacificus* show a bacterium-specific transcriptional response, with relatively few common genes being regulated across multiple bacteria in a given nematode.

### Pfam domain enrichment analysis identifies a role for lipid metabolism and the detoxification machinery in pathogen response in *P. pacificus*

Identification of functional components in large gene-sets such as the pathogen response expression profiles can be better achieved by meta-analysis based on functional annotations rather than by searching for a gene-to-gene correspondence. Therefore, we tested for enrichment of various Pfam domains [52] in the proteins corresponding to the differentially expressed genes (see Methods for details) to see if common functional themes emerge despite only partially overlapping gene lists.

For *C. elegans*, we find the domain enrichment profile to be similar for all bacteria except *S. aureus*. The common domains include those related to Proteasome function, ATPase activity (AAA domain), DNA helicases with DEAD box and Helicase_C domains, and the RRM_1 motif that is indicative of RNA binding protein activity (Table S5). Thus even though the gene-by-gene similarity is low between these expression profiles, we observe proteins with similar functional domains to be enriched in all of them. In *P. pacificus*, expression profiles for all four bacteria are enriched for various lipid metabolism related domains such as Lipase_GDSL, FA_desaturase, Acyl-CoA_dh_1 and Abhydro_lipase (Table S6), suggesting a role for lipid metabolism in *P. pacificus* immune response. Studies on *C. elegans* immune response have shown that the poly-unsaturated fatty acids gamma-linolenic acid and stearidonic acid are integral for immune response, acting via the p38 MAP Kinase pathway [53]. Lipases can potentially function as antagonists of invading pathogens [54] and are known to be induced in response to pathogens in both *C. elegans* [9] and *Drosophila melanogaster* [55], [56]. We also see induction of lipase-like genes *lipl-1* and *lipl-3* across all pathogens in *C. elegans* (Table S1). Thus the enrichment of proteins containing lipase and related domains might contribute towards enhanced resistance of *P. pacificus* on some of the pathogens.

C- type lectins have been implicated in the *C. elegans* innate immune response [57] and in transcriptomic studies of exposure to *P. aeruginosa*, *M. nematophila* and *S. marcescens* [9], [43]–[46], [58]. Although C-type lectin domain encoding genes were differentially expressed when either *C. elegans or P. pacificus* was fed our four pathogens, the enrichment for the corresponding Pfam domain “Lectin_C” achieved statistical significance only in *P. pacificus* profiles on all pathogens except for that on *S. aureus* (Table S6).

The Pfam domains enriched in *P. pacificus* upon response to the relatively less pathogenic bacteria *S. aureus* as well as on exposure to the highly pathogenic *X. nematophila* also include various domains involved in detoxification and xenobiotic defense, such as Glucuronosyltransferase (UDPGT), Glutathione S-transferase (GST_C) and Cytochrome P450 domain (Table S6), which have been previously identified in expression studies of *C. elegans* exposed to xenobiotic compounds [59]. Interestingly, these domains have undergone an expansion in *P. pacificus* genome relative to the *C. elegans* genome and have been hypothesized to have adaptive significance in context of its necromenic lifestyle [18]. Here for the first time we show that the gene activity for the proteins containing these domains is enriched in a potentially pathogenic scenario and possibly confers an adaptive advantage.

Taken together, the Pfam domain analysis provides further insights into the pathogen response of the two nematodes. We observe similar Pfam domains to be enriched within a given nematode in response to different pathogens, but the set of enriched domains differs between *C. elegans* and *P. pacificus*, such that apart from the DNA helicase domain Helicase_C, we hardly find any other domains common between *C. elegans* and *P. pacificus*, even in response to the same bacteria.

### Inhibition of translation machinery is a conserved effect of exposure to gram-negative pathogens in both *C. elegans* and *P. pacificus*

**Figure 5:**
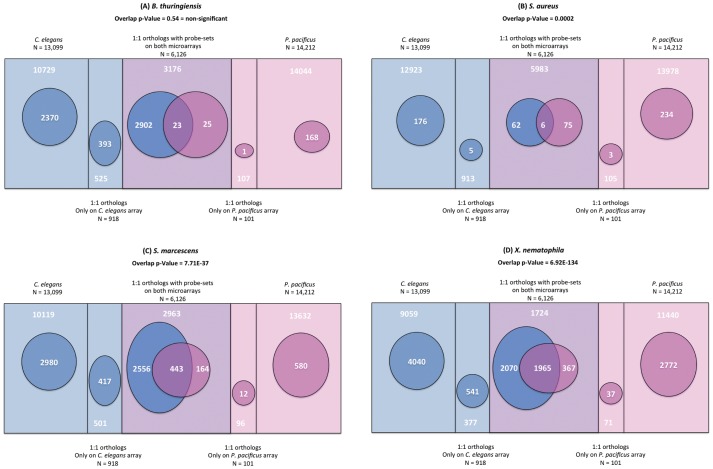
Overlap between *C. elegans* and *P. pacificus* expression profiles in response to different bacteria. The rectangular boxes represent the entire transcriptomes of *C. elegans* and *P. pacificus* genes assayed on our microarrays, and their area of overlap represents the set of 6,126 1∶1 orthologs present on microarrays of both the nematodes. The ovals represent the fraction of differentially expressed genes in each of the subsets. For the 1∶1 orthologs, we assessed the significance of overlap between the genes differentially expressed in response to a given pathogen using a 2×2 Fisher's exact test. Differences or similarities in survival characteristics of the two nematodes when exposed to the same bacteria are reflected in their respective transcriptional responses. (A) On *B. thuringiensis* DB27, which is highly lethal to *C. elegans* but not to *P. pacificus*, only 23 genes are common between the respective expression profiles of the two nematodes, this overlap being statistically not significant. (B) Similarly, *S. aureus* is more lethal to *C. elegans* than *P. pacificus*, and the overlap between the corresponding expression profiles is limited to just 6 orthologs. Although this overlap is statistically significant (p-value = 0.0002), the extent of overlap is too small to be biologically significant. (C) *S. marcescens* is lethal to both the nematodes and the extent and significance overlap between the orthologs differentially expressed in the corresponding expression profiles is also relatively high (443 common orthologs, p-value = 7.71E–37). (D) On the pathogen *X. nematophila*, the observed overlap between the expression profiles in the two nematodes is even higher, with 2,093 orthologs regulated in both nematodes (p-value = 6.92E–134).

Since *C. elegans* and *P. pacificus* have very different survival behavior on the bacteria tested, we wanted to identify genes whose expression might be responsible for these differences. For comparing the expression profiles across the two nematode species, we restricted our analysis to the 6,126 1∶1 orthologous pairs defined by the stringent best reverse BLAST method, for which the probes were present on both the microarrays (see Methods). Interestingly, we see different patterns of overlap between the expression profiles of the two nematodes depending on the bacteria tested. For the gram-positive bacteria *B. thuringiensis* DB27 and *S. aureus*, which kill *C. elegans* at a much higher rate than *P. pacificus*, we observe a very limited overlap in the expression profiles of the two species (Figure 5A and Figure 5B). It was *a priori* not clear whether the *P. pacificus* resistance to *B. thuringiensis* and *S. aureus* is due to induction of similar genes as in *C. elegans*, albeit at higher expression levels, or, if the activation of a totally different set of genes causes the resistance phenotype. The surprisingly small extent of overlap observed in our comparisons supports the second model. Since most of the genes induced in *P. pacificus* on gram-positive bacteria do not have a characterized function yet, future studies will shed light on their role in innate immunity.

In contrast, we observe a highly significant overlap between the *P. pacificus* and *C. elegans* expression profiles in response to the gram-negative bacteria, *S. marcescens* and *X. nematophila*, which are lethal to both the nematodes (Figure 5C and Figure 5D). This suggests that either these gram-negative bacteria induce a similar immune response in both the nematodes, or that late and secondary markers of pathogenesis dominate the expression profile related damages in both the nematode species.

Exposure to pathogens can be expected to affect germline development and reproduction, which might contribute to the set of differentially expressed genes. Consistent with this expectation, we see enrichment of oocyte and germline related expression clusters [60], [61] in both *C. elegans* and *P. pacificus* profiles on various pathogens (see Tables S3 and S4, clusters “cgc6390:oogenesis-enriched”, “WBPaper00037611:RNP-8-associated” and “WBPaper00037611:GLD-2-associated”). The overlap is strongest with the genes downregulated in response to the most lethal pathogens *S. marcescens* and *X. nematophila*.

To identify the conserved elements of the pathogen response in the two species, we focused on the gram-negative pathogens *S. marcescens* and *X. nematophila*, which are lethal to both the nematodes. Almost all the genes common between the two nematodes (Figure 5C and Figure 5D) show a downregulation in response to each of these bacteria (Tables S7A and S7B). We further found 410 genes to be common across both two nematodes on both the gram-negative pathogens, most of them being downregulated (Table S7C). Interestingly, the stress responsive transcription factor encoded by *pqm-1* was differentially expressed across all the four expression profiles (Table S7C), suggesting a potential and conserved role in innate immunity [43] across the two nematode species. Gene Ontology based analysis (Table S8) on any of these three lists shows an enrichment for biological processes related to “determination of adult lifespan”, as well as processes related to protein translation such as “translational elongation”, “translational initiation” and “ribosome biogenesis”. The corresponding terms under the ontology molecular function include “structural constituent of ribosome”, “translation elongation factor activity”, and “translation initiation factor activity”, and the enriched “cellular component” terms include “ribosome”, “ribonucleoprotein complex” and “small ribosomal unit”. Interestingly, it has recently been shown that inhibition of essential cellular processes like translation activates pathogen defense in *C. elegans* [62] and the gram-negative pathogen *Pseudomonas aeruginosa* response in *C. elegans* is activated by detecting its inhibitory effects on translation machinery [63], [64]. Downregulation of components of translational machinery and ribosomes was also observed upon germline-ablation of *P. pacificus* that exhibited an increase in life-span as well as resistance to the pathogen *S. marcescens* [66]. Hence our data together with these recent studies suggest that that downregulation of translation machinery could be a conserved response across the two nematodes, at least when exposed to gram-negative pathogens. We also find enrichment for genes involved in “proteasome complex” and “nuclear pore” complex, and these cellular components are known to have a potential role in immune response in *C. elegans* and *P. pacificus* longevity and immunity [62], [65], [66]. Other enriched processes commonly affected across the two nematodes include various processes related to metabolism, such as “glycolysis”, “tricarboxylic acid cycle” and “fatty acid metabolic process” (Table S8) and cellular compartment GO terms such as “mitochondrial membrane” and “mitochondrial proton-transporting ATP synthase complex” (Table S8). These results suggest that exposure to a pathogens leads to similar changes in the metabolic activity of the two nematodes.

It should also be noted that apart from the overlap between *C. elegans* and *P. pacificus*, differential expression of a substantial number of 1∶1 orthologs is specific to each of the nematode species. Additionally, the portion of the transcriptome with unresolved or no sequence similarity across the two species (Figure 5, rectangular areas specific to either *C. elegans* or *P. pacificus*) is also a significant contributor to the transcriptional response to the pathogens. Hence, based on these analysis of genes in the two nematodes exposed to the same gram-negative bacteria, it seems reasonable to conclude that some effectors of innate immunity are conserved across the two nematodes while some have diverged considerably during the last 250–300Mya separating the two nematode lineages.

### Expression cluster based analysis identifies role for DAF-16, TGF-beta and p38 MAP Kinase pathways in pathogen response

To identify potential upstream regulators of immune response in the two nematodes, we tried using the existing knowledge from *C. elegans* to investigate what pathways appear to be mis-regulated. We therefore assessed the significance and the extent of overlap of our gene-sets with published microarray data sets available as “Expression Clusters” from WormBase [67] as well as with manually curated gene expression data from published microarray studies that were not available in WormBase (see Methods for details). These annotations were transferred to *P. pacificus* genes via the 1∶1 orthology relations.

In agreement with the role of DAF-16 in innate immunity [68], [69], we find DAF-16 targets to be enriched in genes up-regulated in *C. elegans* response to all pathogens except *B. thuringiensis* DB27 (cluster Murphy_etal_cgc5976_Class1 in Table S3B), while the DAF-16 repressed genes are over-represented in the set of down-regulated genes on *B. thuringiensis* and *S. aureus* (cluster Murphy_etal_cgc5976_Class2, Table S3B). Similarly, TGF-beta targets regulated by the ligand DBL-1 [70] are also enriched in many of our expression profiles (clusters “Roberts_etal_2010_DBL-1-UP” and “Roberts_etal_2010_DBL-1-DOWN”, Table S3B), confirming an important role of TGF-beta pathway in response to specific pathogens [9], .

Different MAP kinase pathways such as p38 MAPK and JNK pathways play a key role in *C. elegans* innate immunity and stress response [12], [49]. Consistent with this, we also see a significant overlap with genes regulated by the MAPKK SEK-1 and the JNK-like MAPK KGB-1, especially with down-regulated genes in all pathogen profiles (e.g cluster “Kao_etal2011_sek1_regulated”, Table S3B). We also observe a robust induction of starvation response genes [71] (e.g. cluster “WBPaper00032948:StarveUp3”, Table S3C) within just 4 hours of pathogen exposure, a reasonably short time not expected to induce actual starvation. This observation highlights the importance of metabolism related pathways in immune response [46], [72]–[75]. We also observe enrichment of various dauer related gene clusters and other clusters regulated by stress such as heat shock and oxidative stress in some of the *C. elegans* profiles (Table S3C).

Somewhat similar patterns of overlaps with *C. elegans* expression clusters are also seen for *P. pacificus* pathogen response profiles (Table S4). Interestingly, unlike *C. elegans*, the clusters of genes regulated in response to Cry5B toxin and KGB-1 or SEK-1 MAP kinases show a significant overlap only with genes down-regulated upon exposure of *P. pacificus* to *S. marcescens* and *X. nematophila* (e.g. cluster “Kao_etal2011_sek1_regulated”, Table S4B) but not with genes up-regulated in *P. pacificus*. This suggests potential differences either in targets of the MAPK pathways, or differences in mechanism of activation of these pathways, which might account for enhanced resistance of *P. pacificus*.

*C. elegans* DAF-16 targets are enriched in some *P. pacificus* profiles (cluster “Murphy_etal_cgc5976_Class2” in Table S4B), suggesting that DAF-16 might have a conserved role in innate response in both the species, at least on some pathogens. Interestingly, compared to *C. elegans* profiles, all *P. pacificus* profiles show a significant and more extensive overlap with genes involved in osmotic stress response [76] (e.g. cluster “WBPaper00035873:osmotically_regulated”, Table S4C), suggesting that osmotic regulation could be an important survival mechanism against potentially pathogenic bacteria [76].

### Differential expression of *P. pacificus* pioneer genes

About 30% of the predicted transcriptome of *P. pacificus* is comprised of “pioneer genes”, which do not show any detectable homology to the known protein universe [19] and whose functions are not known. We investigated their potential role in pathogen response by looking at their expression data. We indeed find 832 of these pioneer genes to be differentially expressed *P. pacificus* in a pathogen specific manner, with 160 genes being regulated on at least two pathogens (Figure S3 and Table S9). On each of the pathogens, the pioneer genes constitute 12% to 18% of the active transcriptome, significantly less than the expected proportion of about 30% (Fisher's 2×2 exact test p –values<0.001, Figure S4). Interestingly however, we find the differential expression levels of these pioneer genes to be significantly higher than the non-pioneer fraction of the respective transcriptomes (Figures S5A, S5B, S5C and S5D, Kolmogorov-Smirnov test p-values less than 2.00E–16 for all four pathogens), indicating specific increase in their expression levels after exposure to pathogens. These observations together suggest that some of these lineage specific genes might have been acquired for adaptation to a microenvironment populated by different set of bacteria, some of which might be pathogenic. We can thus ascribe a putative role for these pioneer genes in pathogen response, although further studies will be needed to test these predictions.

### An ecological perspective on the evolution of effectors and regulators of nematode immunity

Our finding that *C. elegans* mounts a pathogen-specific transcriptional response is in agreement with the current understanding in the field [46], [47], [58]. We further show for the first time that the nematode *P. pacificus* can also activate a pathogen-specific response. Many evolutionary mechanisms contribute towards generating this specificity in invertebrates, which lack an adaptive immune system. These include high genetic diversity receptors and effectors involved in pathogen recognition [77], evolutionary diversification of innate immunity effectors e.g. C-type lectins [57], lysozymes [78] and *nlp*- family of antimicrobial effectors [79], natural variation in host susceptibility and virulence of the pathogen [35], [38], [80], and evolution of mechanisms such as recombination and sexual reproduction [81] or alternative splicing [82], [83], all of which facilitate generation of genetic diversity.

The ecology of the organism is expected to be one of the key driving forces behind these changes, as the related species or even strains that occupy different ecological niches will be exposed to different set of non-pathogenic and pathogenic microbes and will need different strategies to survive. Due to these differences in selective pressures, the effectors of their immune systems can be expected to diverge rapidly and also affect the evolution of the host genomes.

The differences observed between response of *C. elegans* and *P. pacificus* can thus be best explained in the light of the distinct ecological niches occupied by both species. While *C. elegans* has recently been isolated from rotting fruit [28], *Pristionchus* nematodes and *P. pacificus* have a strong association with scarab beetles [29]–[32]. Once the beetle dies bacteria proliferate on the rotting carcass allowing mass growth of *Pristionchus* nematodes. Using a metagenomic approach we have previously shown that hundreds of plant and animal pathogenic bacteria occur on and in *Pristionchus* nematodes emerging from beetles [34]. Thus, *Pristionchus* is naturally exposed to a variety of bacteria and has evolved mechanisms to combat infections. Relative to *C. elegans*, the *P. pacificus* genome contains a larger set of genes encoding for cytochrome P450 and UDP-glucoronosyl/UDP-glucosyl transferases, which are required for coping with xenobiotic compounds [18], and we show here that differential activation of these gene families might contribute to its higher resistance to pathogens. The limited overlap on a gene-by-gene between expression profiles on different pathogens combined with the observation that similar Pfam domains are enriched within a nematode species, are consistent with evolutionary diversification and expansion of genes containing these functional domains.

### Conclusions

This study provides a system wide analysis of the transcriptomic responses of the two nematode model species *C. elegans* and *P. pacificus* when feeding on four well-characterized bacterial pathogens. Studies on natural variation in the response of *C. elegans* to pathogens have contributed to micro-evolutionary studies of evolution of innate immunity. By adding studies on host-pathogen interactions in *P. pacificus*, we have tried to extend the evolution of innate immunity towards a macro-evolutionary perspective. We have studied nematode response as early as four hours after exposure to bacteria in order to capture initial events. While many previous studies have looked at various time points (4 h, 8 h, 24 h etc), it is known that by this time a common host necrotic response sets in [46]. The data generated for *P. pacificus* is the first of its kind, whereas our *C. elegans* dataset overlaps, in part, with previous studies. We performed these experiments *de novo*, rather than taking data from the literature, to rule out the effect of differences due to the microarray platform. Our *C. elegans* dataset is however, in strong agreement with existing datasets (e.g. [44], [46], [47]. This study fulfilled three major aims. First, it presents a platform for comparative systems biology analysis of two nematode model species. Second, it generates a catalog of genes involved in the evolution of nematode immunity and finally, it identifies pathogen-specific as well as pan-pathogen, conserved responses in the two nematode species.

Research on *C. elegans* and its interactions with bacteria has lead to the identification of several pathways involved in innate immunity [84]. By using an alternative nematode model we have expanded on this knowledge and shown conservation as well as divergence in the transcripts regulated during an immune response when fed different pathogens. Our systematic comparisons of nematode survival and gene expression on multiple pathogens highlights the fact that substantial differences exist in the repertoire of genetic components deployed in response to varied pathogens between *C. elegans* and *P. pacificus*. The resulting catalogs of pathogen specific and pan-pathogen genes provide an entry point to study the mechanism and evolution of individual response genes in future studies. Using expression cluster analysis we could show that homologs of known targets of FOXO/DAF-16, TGF-beta and p38 MAP kinase pathways in *C. elegans* are also significantly enriched in *P. pacificus*, suggesting that the key signaling pathways might regulate innate immunity in both the species. Given the lack of corresponding mutants in the relatively new model system *P. pacificus*, future studies are needed to test this hypothesis.

Evolutionary studies will have to involve more closely related species and strains given the strong differences observed for *C. elegans* and *P. pacificus.* From a *P. pacificus* perspective, more careful analyses of individual genes in additional strains and closely related *Pristionchus* species will be necessary to obtain insight into the evolution of immunity-related gens. Finally, these results argue for the importance of a comparative approach towards uncovering mechanistic details of the genetic basis that accounts for the cross-species variation in susceptibility to a given pathogen.

## Materials and Methods

### Strains

*C. elegans* N2 and *P. pacificus* RS2333 were maintained on NGM plates seeded with *E. coli* OP50. *S. marcescens* was isolated from La Reunion, *S. aureus* Newman was purchased from the Deutsche Sammlung von Mikroorganismen und Zellkulturen, Germany (DSMZ), *X. nematophila* was a gift from Becker Underwood, U.K. *B. thuringiensis* DB27 was isolated from dung beetles [35] and was initially thought to be a strain of *B. cereus* but further sequence analysis has shown it is in fact a strain of closely related *B. thuringiensis*.

### Assessing survival of *C. elegans* and *P. pacificus* exposed to pathogens

Each bacterium was grown in a shaking incubator at 30°C overnight in LB, apart from *S. aureus*, which was grown at 37°C. The following day 100 μl were spread onto previously dried 6 cm NGM plates and incubated overnight. Twenty adults of C. elegans or *P. pacificus* were separately placed onto 3 NGM plates per bacterium and stored at 25°C where survival was monitored daily for 7 days. The experiment was repeated twice. Pathogen survival was compared to worms cultured on *E. coli* control plates and differences in survival were analyzed using Kaplan Meier and logrank test.

### RNA collection for microarray experiments

Synchronized populations of *C. elegans* or *P. pacificus* were obtained by hypochlorite treatment and allowed to grow to young adult stage on *E. coli* OP50 plates at 20°C. For each biological replicate, about 200 young adult hermaphrodites were picked onto pathogen plates for 4 hours of pathogen exposure, after which they were collected into 1mL of TRIzol (Invitrogen). Equal number of corresponding age-matched control worms were exposed to *E. coli* OP50 for 4 hours and transferred to 1ml TRIzol (Invitrogen). Four biological replicates were collected for each experimental and control condition. Total RNA was extracted using TRIzol reagent (Invitrogen) according to manufacturer's instructions. The isolated RNA was further purified using phenol: chloroform: isoamyl alcohol precipitation to remove trace left-overs of TRIzol etc. which might interfere with downstream reactions. The RNA pellet was suspended in RNAse free water and was assessed on a Nanodrop spectrophotometer for quantity and RNA quality. RNA samples were stored at −80°C until the microarray experiments.

### Microarray experiments

A total of 32 microarray hybridizations were carried out for 8 conditions (2 nematode species x 4 pathogenic bacteria; 4 biological replicates per condition). Oligonucleotide microarrays for *C. elegans* containing ∼43,000 unique probes for ∼20,000 *C. elegans* genes were obtained from Agilent Technologies (NCBI GEO accession GPL10094). For *P. pacificus* experiments, we used our custom designed oligonucleotide microarrays manufactured by Agilent Technologies, which contain ∼93,000 unique probes for the ∼23,000 *P. pacificus* predicted genes (NCBI GEO accession GPL14372, see [22] for design details of custom microarrays). The *P. pacificus* gene sequences are available at http://www.pristionchus.org/download/ and the gene models can be seen in the genome browser at http://www.pristionchus.org/cgi-bin/genome.pl.

Equal amounts of total RNA (500 ng to 800 ng) from four biological replicates of each experimental and control samples was used to produce Cy5 or Cy3 dye labeled cRNA using Quick Amp Labelling Kit (Agilent Technologies Inc., USA) as per manufacturer's instructions. Depending upon the amount of total RNA used, appropriate amounts of positive control RNA (Spike Mix-A and Spike Mix-B, from Agilent Technologies) was added to the mix before reverse transcribing the total RNA, as per manufacturer's instructions. We used the *C. elegans* or *P. pacificus* microarrays in a two-color format where Cy5 and Cy3 dye labeled cRNA from experimental and control sample is co-hybridized on the same microarray. The four biological replicates per experiment included two dye-swaps experiments to account for differences in dye labeling. Hybridization and washing of the arrays was carried according to manufacturer-supplied protocol. The arrays were scanned on a GenePix 4000B Microarray Scanner, and raw data extracted using GenePix Pro sofware (version 6).

### Microarray data analysis

We used the Bioconductor [85] package limma [86] for analysis of our microarray data. Array quality was checked for parameters such as uniform background and foreground intensities over the entire array. The raw signal was background corrected using the normexp method [87] and the arrays were then lowess normalized individually (“normalizeWithinArrays” option), with differential weights assigned to probes and to positive control spike-ins, which are expected to show no fold change [88]. This differential weighing of probes is particularly necessary to account for differences in differences in relative proportion of mRNA versus total RNA, and/or differences in the amount of RNA produced per worm under different experimental conditions. Without this differential weighing scheme, the fold change calculations can be erroneous [22], [42]. The weight parameters were optimized based on MA-plots such that spike-in controls show their expected fold change values. lmFit function was used to fit a linear model to probe intensities across arrays, differential expression was calculated by empirical Bayes method using the eBayes function [89], and control of FDR was employed as the multiple testing correction. MA-plots were also used as diagnostic to identify and remove outlier arrays before fold-change calculations, such that at least three biological replicates were used for each experiment. Genes with a FDR corrected p-value less or equal to than 0.05 and absolute log2 of fold change greater than 0.5 were called significantly differentially expressed. Further data analysis was carried out using custom scripts in Perl and the statistical package R. Venn diagrams were drawn using the R package VennDiagram [90]. Raw and processed data from all the experiments from this publication have been deposited in a MIAME compliant format [91] at NCBI's Gene Expression Omnibus database (http://www.ncbi.nlm.nih.gov/geo/). The accession numbers for *C. elegans* data are GSE36413, GSE36493, GSE36499, GSE36501, and the accession numbers for *P. pacificus* data are GSE36517, GSE36519, GSE36521 and GSE36523.

### Identifying 1∶1 orthologs between *C. elegans* and *P. pacificus*

We have previously used a pairwise best BLASTP strategy to identify 7,176 pairs of 1∶1 orthologs between the *C. elegans* and *P. pacificus* [22]. Briefly, all protein sequences from *C. elegans* were run as query versus the database of *P. pacificus* gene predictions and vice versa. Only hits with a BLAST score > = 50 bits were retained, and mutually best hits were identified as 1∶1 orthologs. Probes for 6,126 of these gene pairs exist on microarrays of both the species.

### Pfam domain annotation and enrichment analysis

Pfam domain annotations for *C. elegans* and *P. pacificus* proteomes (WS220 and predicted proteins respectively) were the same as described before [22]. Basically, hits with a p-value cut-off of 0.001 in HMM searches using HMMer 3.0 [92] on Pfam release V23/4 [52] were used as domain annotations. Only the domains, for which minimum 5 protein coding genes were represented on each microarray, were used for further enrichment analysis. Statistical significance of enrichment of Pfam domains in each expression profile determined using a 2×2 Fishers exact test, at a FDR corrected p-value cut-off of 0.05.

### Expression cluster enrichment analysis

We have used “expression cluster” annotations from WormBase [67] in interpretations of other microarray expression profiles [22]. The list of microarray expression profiles in which a given *C. elegans* gene is known to be differentially expressed can be extracted from the section “Expression Cluster” from the WormBase gene summary page for each gene. We retrieved all available expression clusters for *C. elegans* genes from the WormBase web site. We also compiled data from other gene expression studies in *C. elegans* which are relevant to pathogen response but for which the corresponding expression clusters were not available in the WormBase (viz. [12], [49], [70]) and named these clusters with a prefix based on first author's last name and year of publication of the research article, and included them in our analysis. We inferred expression clusters for *P. pacificus* based on the set of 1∶1 orthologs. P-values for expression cluster enrichment in each expression profile was computed with a 2×2 Fisher exact test. FDR corrected p-value cut-off of 0.05 was used as the significance threshold. The significance score was calculated as –log10 of the p-values and was set to zero to indicate non-significance when p-values was greater than 0.05.

### Gene ontology analysis, prediction of signal peptide and antimicrobial activity

Gene ontology analysis (presented in Table S8) was done using Bioconductor tool topGO, using method “elimFisher” for calculating p-values [93]. For analyzing features of differentially expressed pioneer genes in *P. pacificus* (Table S9), SignalP tool was used to predict the presence of a signal peptide [94], and for genes coding for products smaller than 100 amino acids, CAMP tool [95] was used to predict whether they can act as potential Anti-Microbial Peptides (AMPs).

## Supporting Information

Figure S1 **Long-term survival curves for** ***P. pacificus*** **on various pathogens.** *P. pacificus* has higher resistance than *C. elegans*, with longer median survival time of about 8 days on *B. thuringiensis* and *S. aureus*.
(PDF)

Figure S2 **Global transcriptional suppression in response to pathogens.** Exposure to pathogens resulted in a decrease of amount of total RNA produced per worm when compared to the non-pathogenic *E. coli* strain. This global decrease in transcription is seen in both (A) *C. elegans* and (B) *P. pacificus*.
(PDF)

Figure S3 **Overlap between pioneer genes regulated in** ***P. pacificus*** **in response to the four pathogens**. Of the 832 pioneer genes differentially expressed on any of the pathogens in *P. pacificus*, 160 genes are common between two or more than two expression profiles.
(PDF)

Figure S4 **Relative proportions of pioneer genes versus non-pioneer genes in the active transcriptome of** ***P. pacificus*** **on each of the four pathogens.** The *P. pacificus* genome contains about 30% pioneer genes. Compared to the random expectation of the same proportion of pioneer genes in different expression profiles, they are found to significantly under-represented (Fisher's test p-values<2E-16 for each comparison with whole-genome distribution.
(PDF)

Figure S5 **Pioneer genes are expressed at higher levels than non-pioneer genes in each of the pathogen-induced expression profiles on** ***P. pacificus*** **.** Cumulative distributions of fold-changes of pioneer genes (red curves) and non-pioneer genes (blue curves) for genes differentially expressed on (A) *B. thuringiensis* DB27 (B) *S. aureus* (C) *S. marcescens* and (D) *X. nematophila*. The Kolmogorov-Smirnov test p-values are less that 2E-16 in each case.
(PDF)

Table S1 **Differential expression of genes in** ***C. elegans*** **exposed to different pathogens.**
(XLSX)

Table S2 **Differential expression of genes in** ***P. pacificus*** **exposed to different pathogens.**
(XLSX)

Table S3 **Expression clusters enriched in genes up- and down-regulated in** ***C. elegans*** **upon exposure to each of the four pathogens.**
(XLSX)

Table S4 **Expression clusters enriched in genes up- and down-regulated in** ***P. pacificus*** **upon exposure to each of the four pathogens.**
(XLSX)

Table S5 **Pfam domains enriched in** ***C. elegans*** **expression profiles in response to different pathogens.**
(XLSX)

Table S6 **Pfam domains enriched in** ***P. pacificus*** **expression profiles in response to different pathogens.**
(XLSX)

Table S7 **Genes common across expression profiles of** ***C. elegans*** **and** ***P. pacificus*** **upon exposure to the gram-negative bacteria (A)** ***S. marcescens*** **(B)** ***X. nematophila*** **, and (C) common to both** ***S. marcescens*** **and** ***X. nematophila*** **expression profiles.**
(XLSX)

Table S8 **GO enrichment analysis of genes common across both** ***C. elegans*** **and** ***P. pacificus*** **profiles obtained upon exposure to gram-negative bacteria** ***S. marcescens*** **and** ***X. nematophila*** **.**
(XLSX)

Table S9 **Features of pioneer genes differentially expressed on any of the four pathogens in** ***P. pacificus*** **.**
(XLSX)
