# Supplementary material for: System Wide Analysis of the Evolution of Innate Immunity in the Nematode Model Species Caenorhabditis elegans and Pristionchus pacificus
Source: PLoS One. 2012 Sep 28;7(9):e44255. doi: 10.1371/journal.pone.0044255 (PMC3461006; doi:10.1371/journal.pone.0044255)

Supplementary Figure S1 : Long term survival curves for *P. pacificus* on various pathogens.

P. pacificus has higher resistance than *C. elegans* to *B. thuringiensis* and *S. aureus*, seen as longer median survival time of about 8 days on these pathogens

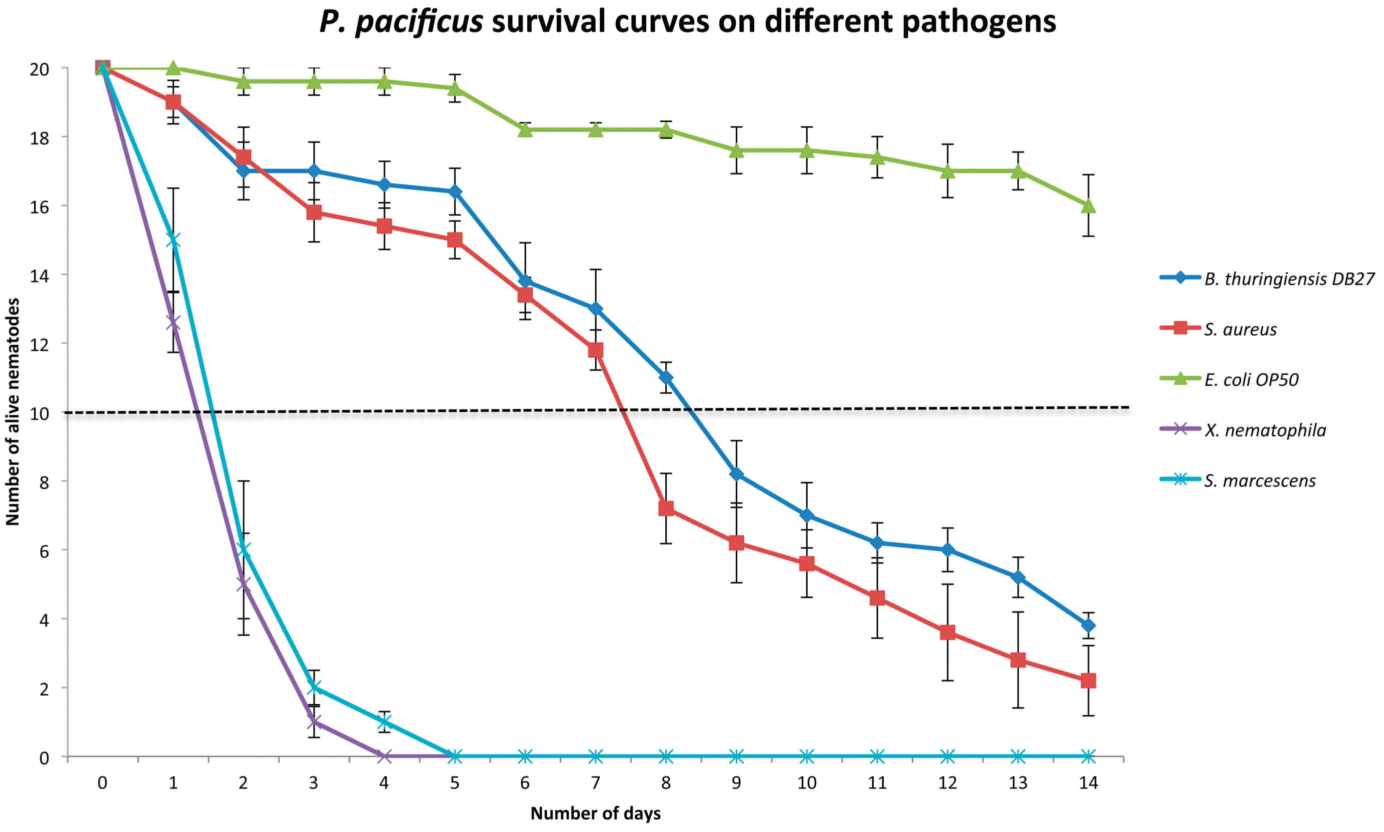

Supplement: Figure S1 — Long-term survival curves for P. pacificus on various pathogens. P. pacificus has higher resistance than C. elegans, with longer median survival time of about 8 days on B. thuringiensis and S. aureus. (PDF) [file pone.0044255.s001.pdf]
