# Supplementary material for: System Wide Analysis of the Evolution of Innate Immunity in the Nematode Model Species Caenorhabditis elegans and Pristionchus pacificus
Source: PLoS One. 2012 Sep 28;7(9):e44255. doi: 10.1371/journal.pone.0044255 (PMC3461006; doi:10.1371/journal.pone.0044255)

Supplementary Figure S2 : Global transcriptional suppression in response to pathogens in (A) *C. elegans* (B) *P. pacificus*

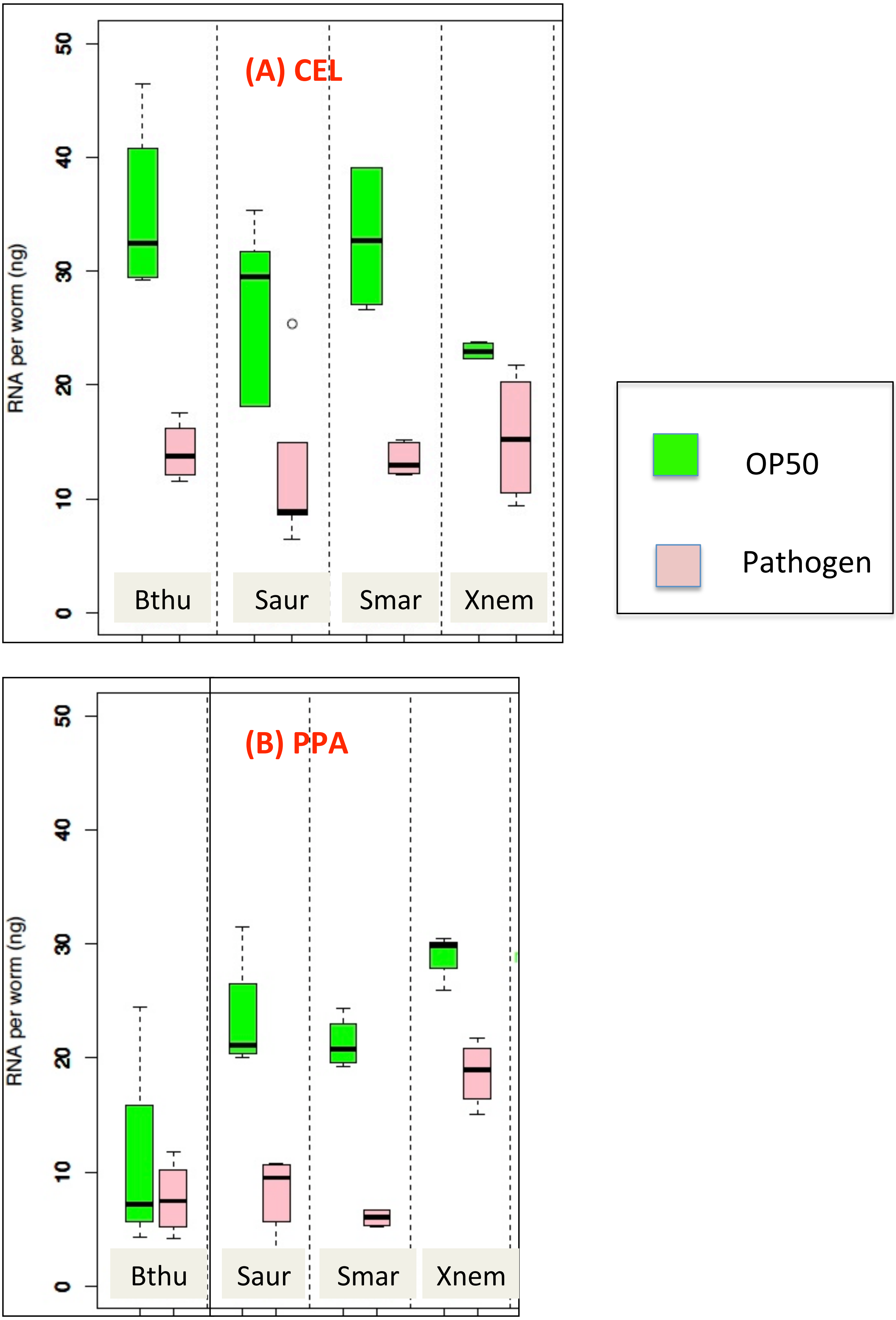

Supplement: Figure S2 — Global transcriptional suppression in response to pathogens. Exposure to pathogens resulted in a decrease of amount of total RNA produced per worm when compared to the non-pathogenic E. coli strain. This global decrease in transcription is seen in both (A) C. elegans and (B) P. pacificus. (PDF) [file pone.0044255.s002.pdf]
