# Supplementary material for: System Wide Analysis of the Evolution of Innate Immunity in the Nematode Model Species Caenorhabditis elegans and Pristionchus pacificus
Source: PLoS One. 2012 Sep 28;7(9):e44255. doi: 10.1371/journal.pone.0044255 (PMC3461006; doi:10.1371/journal.pone.0044255)

Supplementary Figure S3 : Overlap between pioneer genes regulated in *P. pacificus* in response to the four pathogens

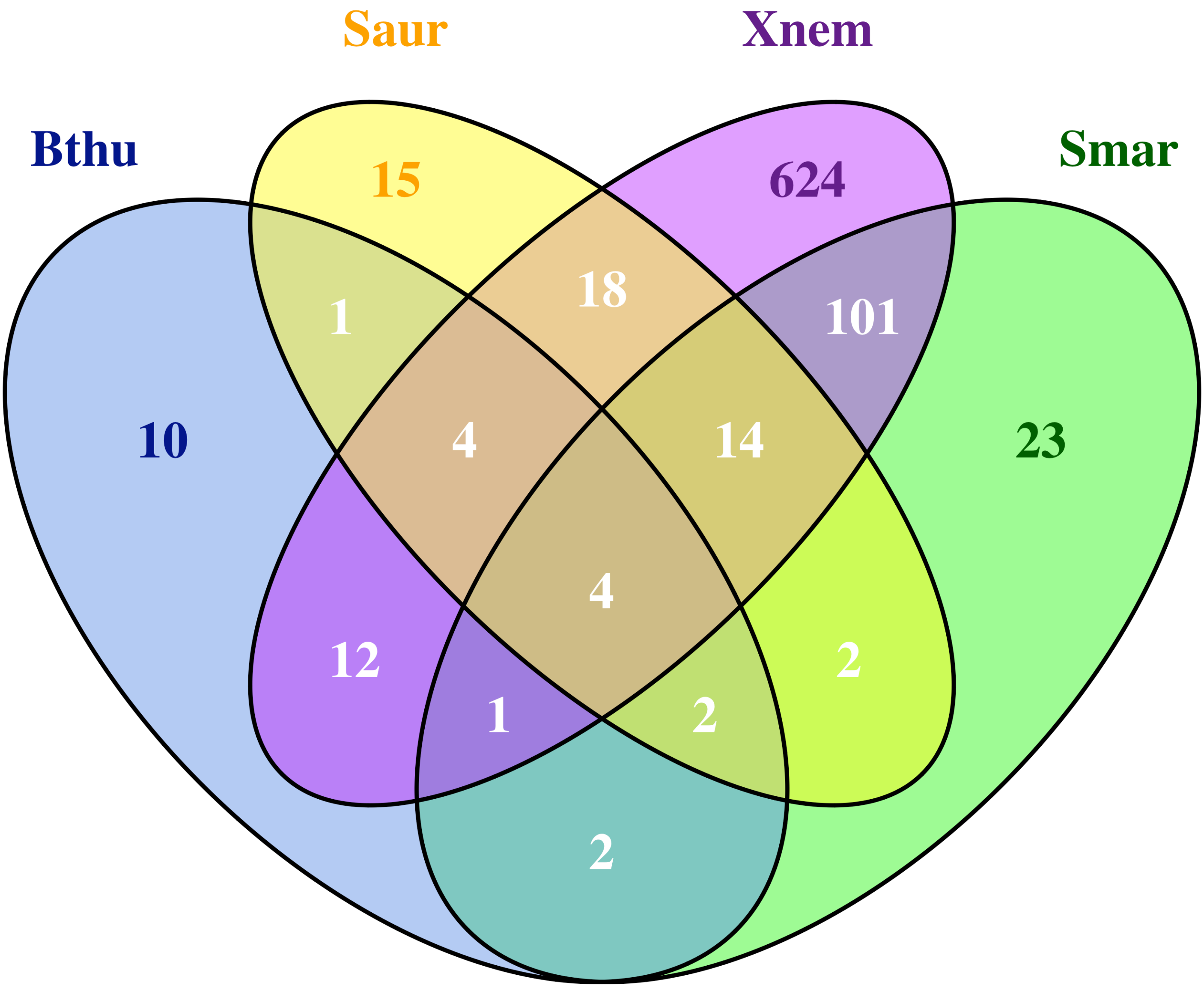

Supplement: Figure S3 — Overlap between pioneer genes regulated in P. pacificus in response to the four pathogens. Of the 832 pioneer genes differentially expressed on any of the pathogens in P. pacificus, 160 genes are common between two or more than two expression profiles. (PDF) [file pone.0044255.s003.pdf]
