# Supplementary material for: System Wide Analysis of the Evolution of Innate Immunity in the Nematode Model Species Caenorhabditis elegans and Pristionchus pacificus
Source: PLoS One. 2012 Sep 28;7(9):e44255. doi: 10.1371/journal.pone.0044255 (PMC3461006; doi:10.1371/journal.pone.0044255)

Supplementary Figure S4 : Relative proportions of pioneer genes versus non-pioneer genes in the active transcriptome of *P. pacificus* on each of the four pathogens.

On each of the pathogens, the pioneer genes constitute 12% to 18% of the active transcriptome, significantly less than the expected proportion of about 30% from the distribution in the entire transcriptome.
One-sided Fisher's 2x2 exact test p –values are (a) 9.72E-05 on *B. thuringiensis* (b) 1.01E-04 on *S. aureus* (c) 8.75E-39 on *S. marcescens*, and (d) 8.01E-128 on *X. nematophila*

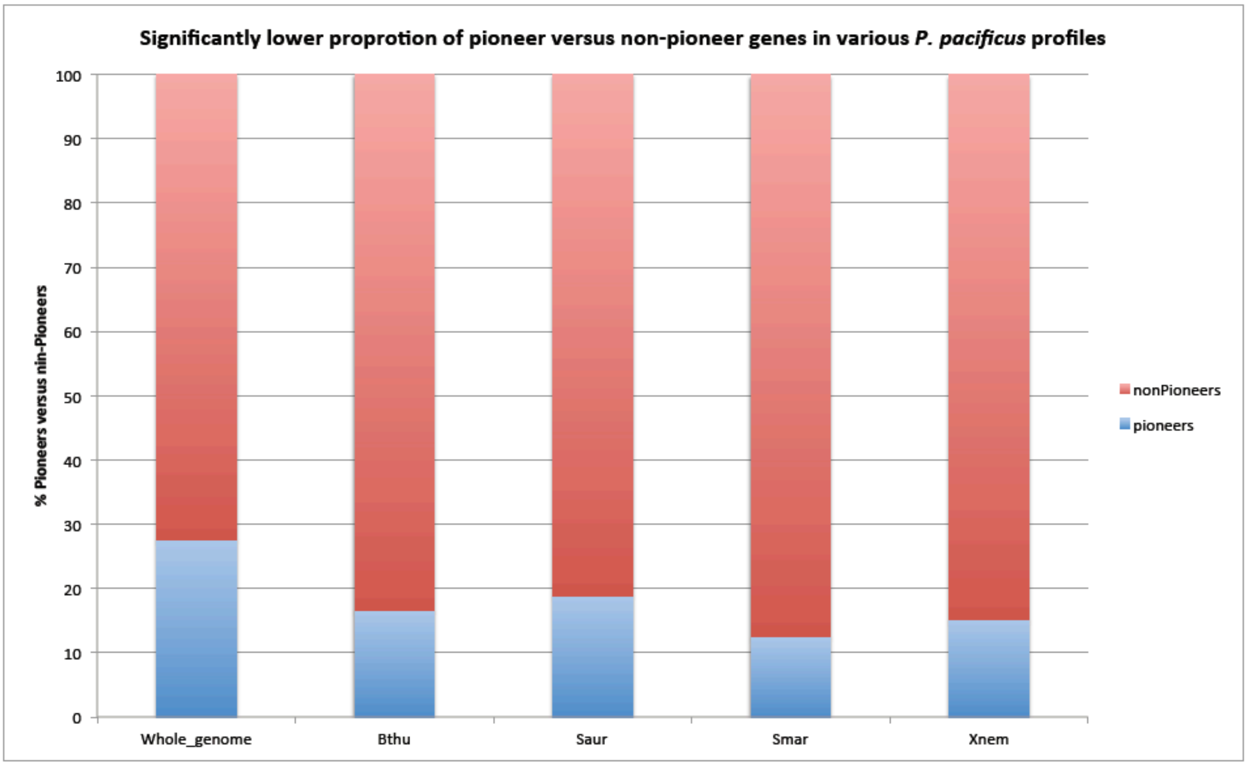

Supplement: Figure S4 — Relative proportions of pioneer genes versus non-pioneer genes in the active transcriptome of P. pacificus on each of the four pathogens. The P. pacificus genome contains about 30% pioneer genes. Compared to the random expectation of the same proportion of pioneer genes in different expression profiles, they are found to significantly under-represented (Fisher's test p-values<2E-16 for each comparison with whole-genome distribution. (PDF) [file pone.0044255.s004.pdf]
