# Supplementary material for: System Wide Analysis of the Evolution of Innate Immunity in the Nematode Model Species Caenorhabditis elegans and Pristionchus pacificus
Source: PLoS One. 2012 Sep 28;7(9):e44255. doi: 10.1371/journal.pone.0044255 (PMC3461006; doi:10.1371/journal.pone.0044255)

Supplementary Figure S5 .Pioneer genes are expressed at higher levels than non-pioneer genes in each of the pathogen-induced expression profiles on *P. pacificus*.

(A) *B. thuringiensis*

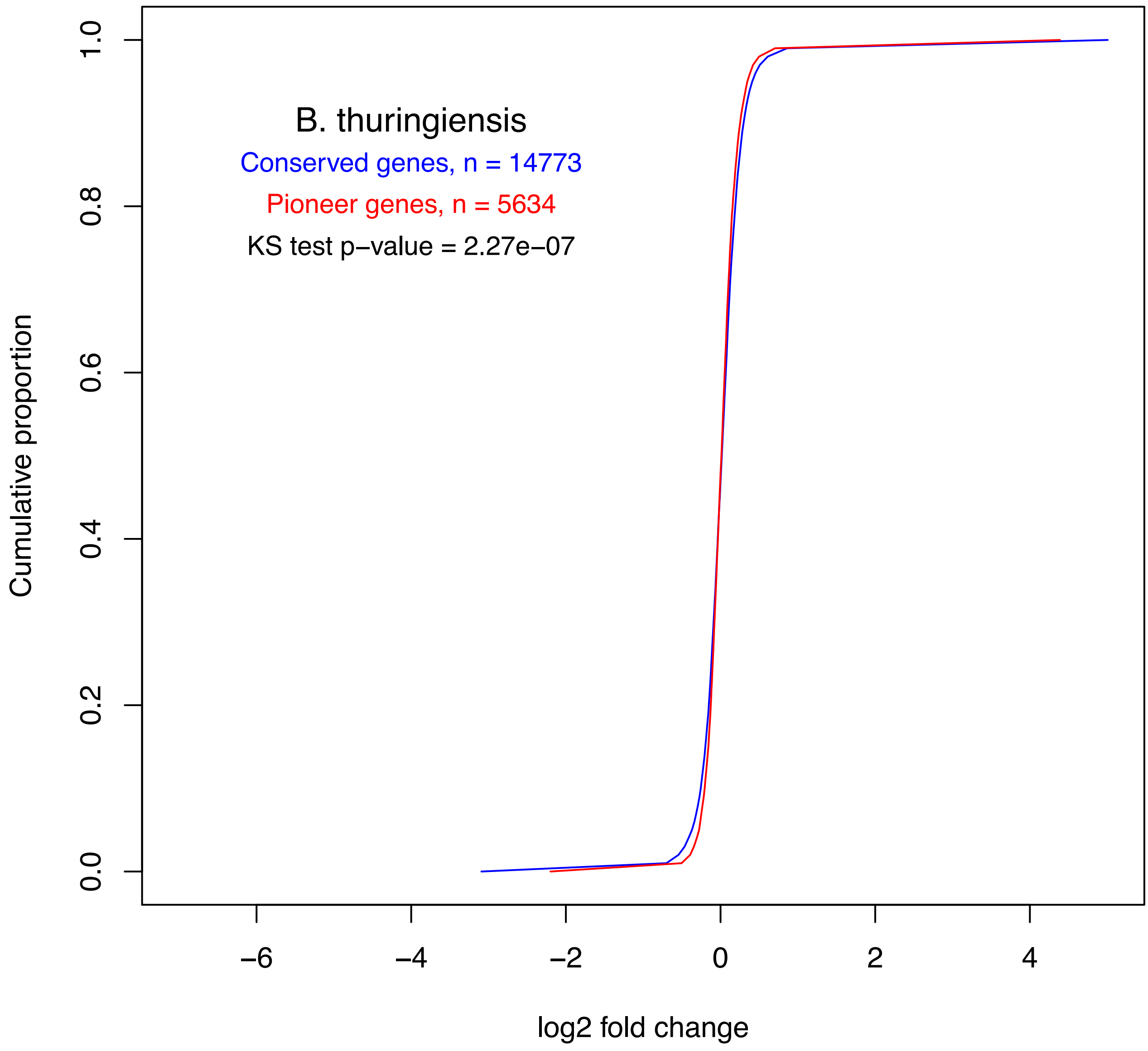

Supplementary Figure S5 .Pioneer genes are expressed at higher levels than non-pioneer genes in each of the pathogen-induced expression profiles on *P. pacificus*.

(B) : *S. aureus*

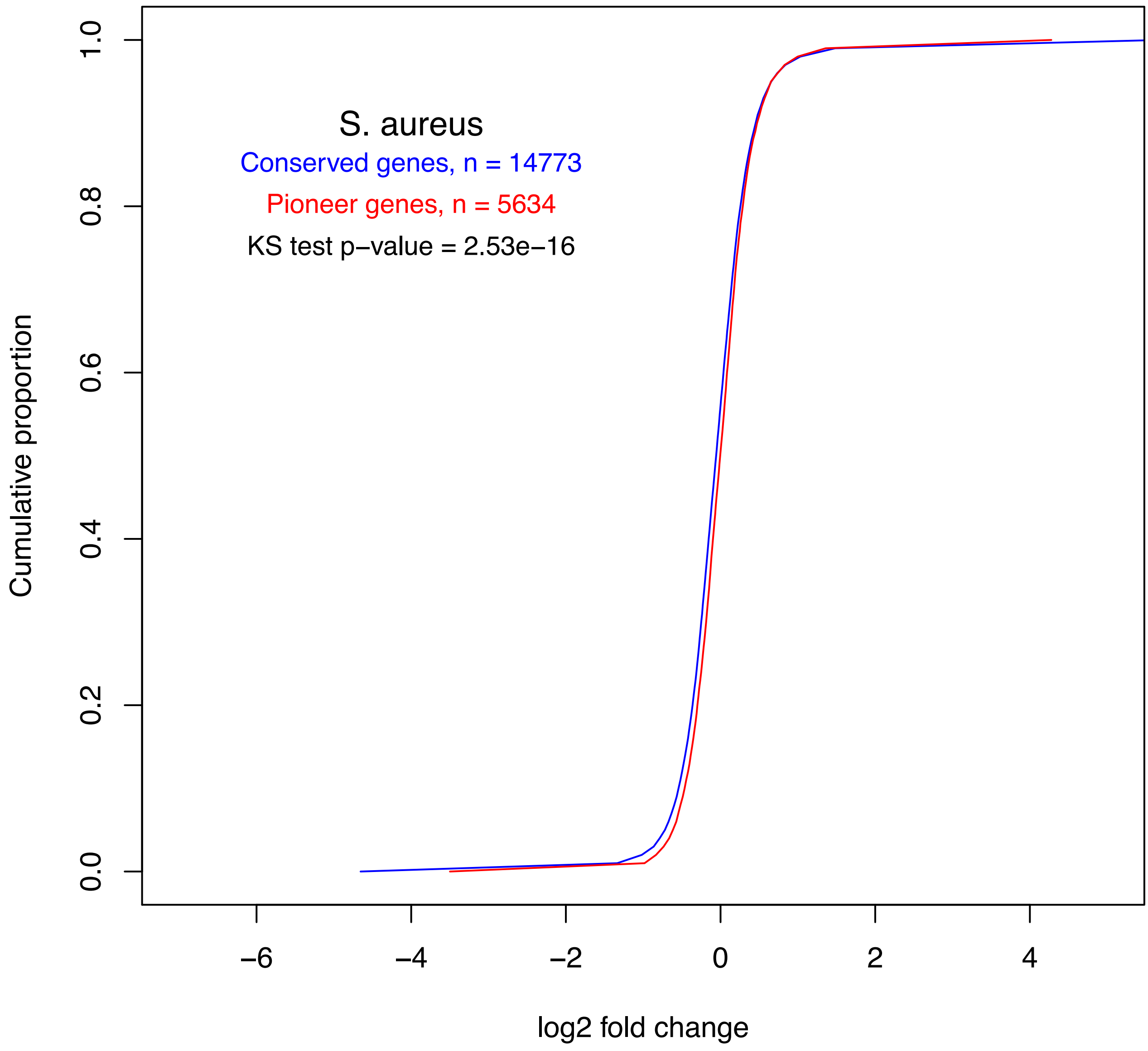

Supplementary Figure S5 .Pioneer genes are expressed at higher levels than non-pioneer genes in each of the pathogen-induced expression profiles on *P. pacificus*.

(C) : *S. marcescens*

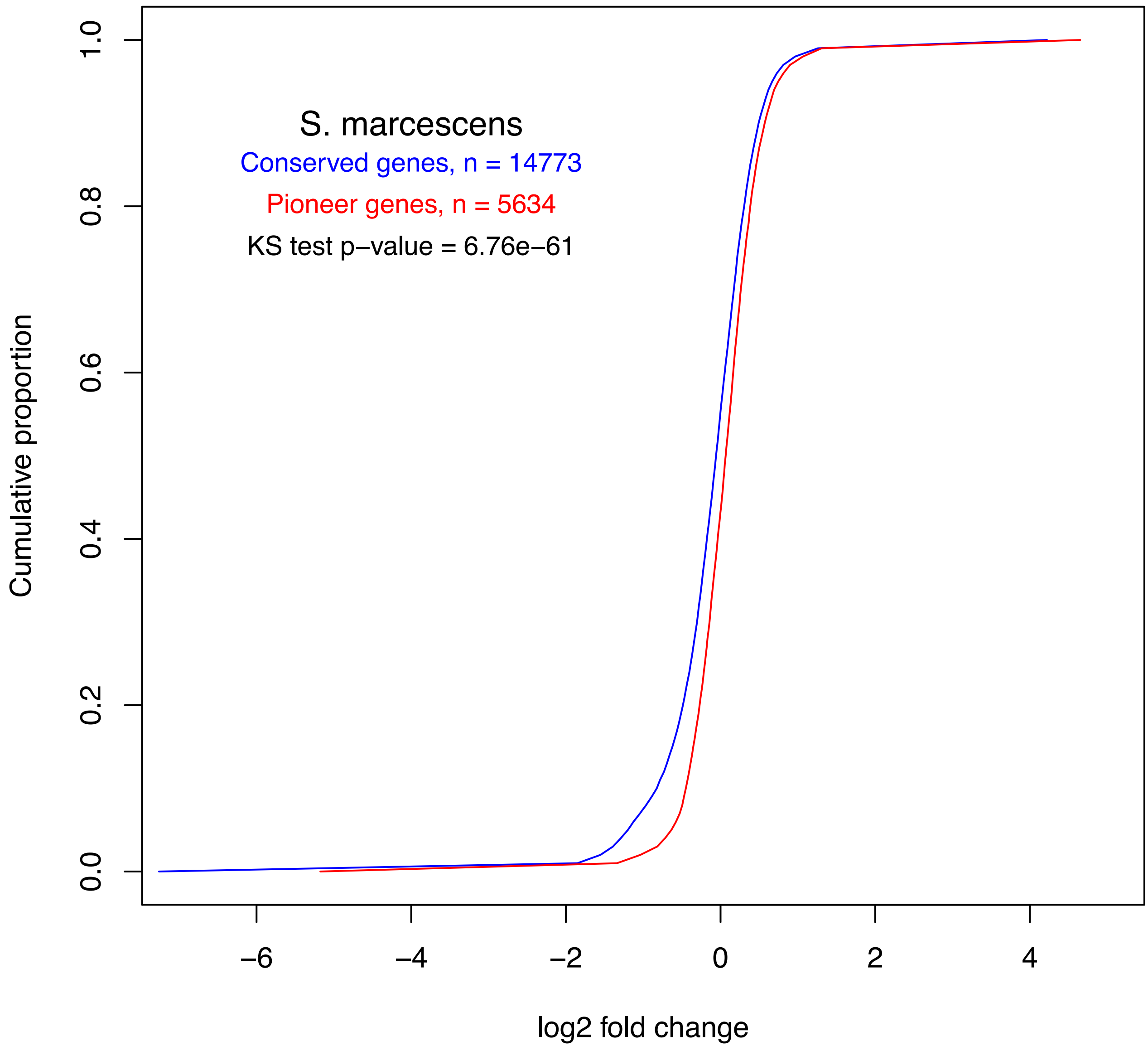

Supplementary Figure S5 .Pioneer genes are expressed at higher levels than non-pioneer genes in each of the pathogen-induced expression profiles on *P. pacificus*.

(D) : *X. nematophila*

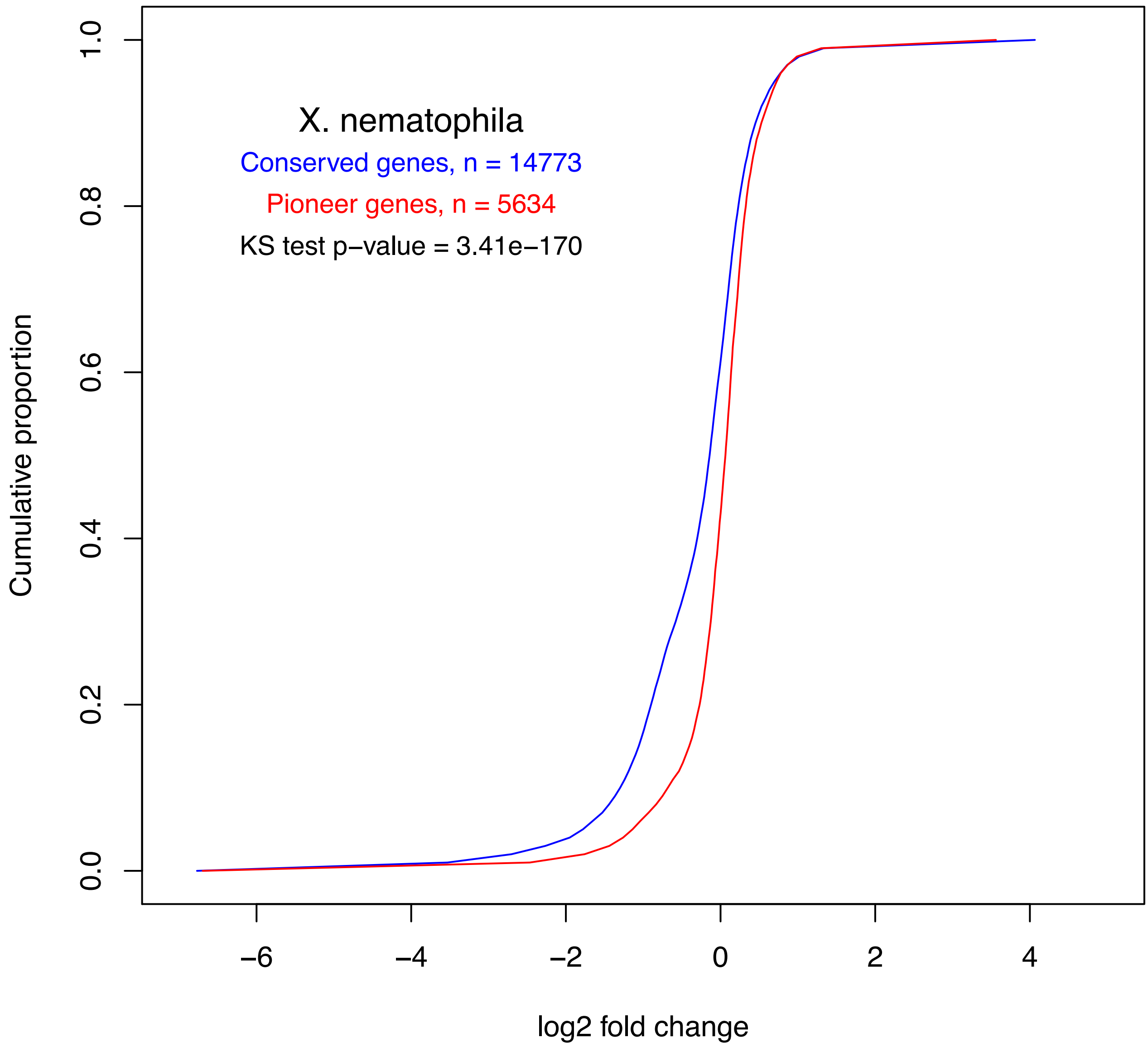

Supplement: Figure S5 — Pioneer genes are expressed at higher levels than non-pioneer genes in each of the pathogen-induced expression profiles on P. pacificus . Cumulative distributions of fold-changes of pioneer genes (red curves) and non-pioneer genes (blue curves) for genes differentially expressed on (A) B. thuringiensis DB27 (B) S. aureus (C) S. marcescens and (D) X. nematophila. The Kolmogorov-Smirnov test p-values are less that 2E-16 in each case. (PDF) [file pone.0044255.s005.pdf]
